# Enhanced phytoplankton bloom triggered by atmospheric high-pressure systems over the Northern Arabian Sea

**DOI:** 10.1038/s41598-023-27785-z

**Published:** 2023-01-14

**Authors:** Prasad G. Thoppil

**Affiliations:** Ocean Sciences Division, Naval Research Laboratory, Stennis Space Center, Stennis Space Center, MS 39529 USA

**Keywords:** Marine biology, Physical oceanography, Atmospheric dynamics, Ocean sciences

## Abstract

During winter, the dry, cool air brought by prevailing northeasterly trade winds leads to surface ocean heat loss and convective mixing in the northern Arabian Sea. The current paradigm is that the convective mixing process leads to the injection of nutrients up into the surface waters and exert a dominant control on winter productivity. By combining a variety of observations, atmospheric reanalysis and model simulations, we unraveled the processes responsible for the observed year-to-year chlorophyll-*a* variations in the northern Arabian Sea. Our findings suggest that the atmospheric high-pressure systems that traverse the northern Arabian Sea every winter and spring disrupt winter convective mixing and create an array of environmental conditions conducive to trigger phytoplankton blooms. The arrival of an atmospheric high with the anticyclonic flow in the northern Arabia Sea sets the stage for a sequence of events culminating in intermittent mixed-layer restratification due to buoyancy gain aided by increased specific humidity, supplemented with abundant sunlight due to clear skies, and suppressed turbulent mixing owing to weak winds. These combined with the mixed layer that is shallower than the euphotic zone and the influx of nutrients into the euphotic zone brought by convective mixing between the calm periods, caused unprecedented high concentrations of chlorophyll-*a* in the northern Arabian Sea.

## Introduction

The primary productivity of the Arabian Sea is governed by seasonally reversing monsoonal winds^1^ and associated physical processes that modulate the upper-ocean^2^. During the summer monsoon (June–September), strong southwesterly winds^3^ drive coastal upwelling along the coasts of Somalia and Arabia which bring nutrients from below and trigger phytoplankton blooms^2,4,5^. During the winter monsoon (November–February) the prevailing northeasterly winds, which bring cold-dry continental air from the north, leads to enhanced buoyancy loss resulting in convective mixing. It is this convective mixing process that is responsible for the injection of nutrients up into the surface waters, and exerts a dominant control on winter productivity^6–11^. However, this mechanism alone cannot explain the recurrent phytoplankton blooms in the northern Arabian Sea during winter. Here we present a sequence of events emanated from the anticyclonic atmospheric high over the northern Arabian Sea leading to wide spread phytoplankton blooms during February–March. The arrival of an atmospheric high-pressure system leads to reversal of winds from northeasterly to southwesterly. In particular, the southwesterly winds to the west of high-pressure system transport warm, humid air from south to the northern Arabian Sea resulting in an increase in specific humidity. The increased specific humidity in turn suppress latent heat flux thereby increasing heat gain by the ocean. This leads to a transient mixed-layer restratification, supplemented with increased sunlight and light winds, resulting widespread winter productivity.

In the following sections, we identify periods of anomalously high phytoplankton blooms and associated mixed layer restratification events and link those events to atmospheric high-pressure systems with a special focus on 2014–2015 period. We then extend the analyses to 2003–2021 periods employing a combination of 1-D model, chlorophyll-*a* observations and atmospheric reanalysis data. The analyses revealed that the mixed layer restratification events appeared to be a recurrent feature in winter and spring and contribute significantly to year-to-year chlorophyll-*a* variability in the northern Arabian Sea.

## Results

### Anomalous Chlorophyll-a blooms

**Figure 1:**
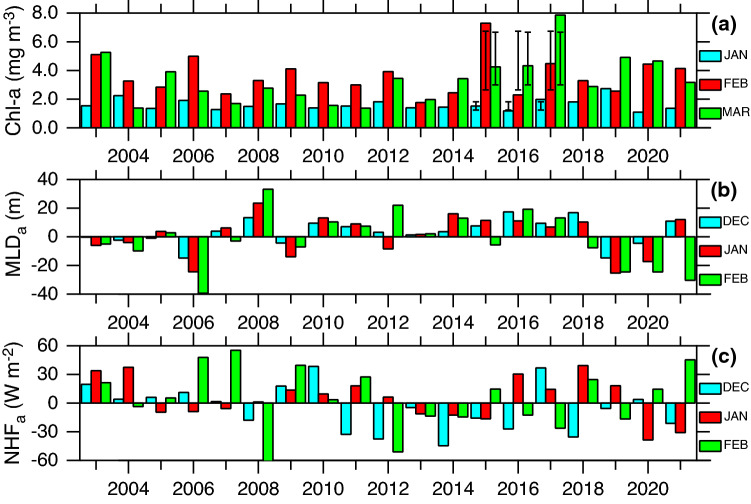
Anomalous chlorophyll-*a* blooms. (**a**) Time-series of monthly mean, area averaged (58°–66° E, 18°–26° N) MODIS-Aqua (2003–2021) chlorophyll-*a* concentration (mg m^−3^) in the northern Arabian Sea during (cyan) January, (red) February, and (green) March. Mean ± standard deviation is plotted for 2015–2017. The anomalous chlorophyll-*a* during 2014–2015 and 2016–2017 exceeds the mean + standard deviation. Anomalies of (**b**) mixed layer depth (m) and (**c**) surface heat flux (W m^−2^) for (cyan) December, (red) January, and (green) February. Mixed layer is defined as the depth at which a density difference of 0.125 kg m^−3^ from the surface using temperature and salinity profiles from EN4.2^32^. Surface heat flux is calculated from CFSR^33^ product. Anomalies are calculated from the 1998 to 2021 mean. The seasonal choice of December–February represents peak mixed layer depth and surface heat flux during winter and phytoplankton bloom during January–March.

In the northwestern Arabian Sea, the winters of 2014–2015 and 2016–2017 experienced by far the highest chlorophyll-*a* concentration (a proxy for phytoplankton biomass) ever recorded in the past two decades of the satellite era (Fig. 1a). The chlorophyll-*a* during these periods exceeded 7 mg m^−3^, over a twofold increase to the long-term (1998–2021) mean. The anomalously high chlorophyll-*a* occurred during February in 2014–2015 winter and during March in 2016–2017 winter. Yet, chlorophyll-*a* concentration during March 2015 and February 2017 were comparable to other years. As a result, the total chlorophyll-*a* concentration during February–March for 2014–2015 and 2016–2017 winter was 11.6 and 12.3 mg m^−3^, an increase of 89% and 102% from the long-term mean, respectively. Despite the anomalous period, the onset of spring bloom, as defined by peak chlorophyll-*a*, typically occurred in February of each year until 2012 with the exception of 2005. However, there has been an increase in spring bloom occurrence in March in the last decade with the strongest event in March 2017 (Fig. 1a). This extended phytoplankton growth can be ascribed to the prolonged winter convective mixing^12^ thereby delaying the onset of vernal restratification, although the exact underlying mechanisms have hitherto been unexplored.

Deep winter convective mixing increases the nutrient input into the euphotic zone, the depth where sunlight is available for photosynthesis, and leads to higher levels of primary productivity^6,8^. Note that convective mixing will export phytoplankton from the euphotic zone to deeper layers but conversely re-supply nutrients from below the productive layer^6,13^. However, anomalies of mixed layer depth (Fig. 1b) during winters of 2014–2015 and 2016–2017 were relatively small and comparable to other years (including 2015–216 with lower chlorophyll-*a*) and therefore cannot entirely explain the unprecedented observed chlorophyll-*a*. The mixed layer anomaly in 2014–2015 winter shows a slightly positive anomaly (deeper mixed layer) during December–January (5–10 m) and shallower mixed layer (5 m) in February. These are consistent with the surface heat flux anomalies (Fig. 1c), which controls the intensity of convective mixing, with excess heat loss during December–January and heat gain in February (10–20 W m^−2^).

Alternately, circulation in the northwestern Arabian Sea including the Gulf of Oman during winter is dominated by mesoscale eddies (resulting from baroclinic instability from the summer that preceded the winter) and submesoscale eddies. However, the mesoscale eddies during 2014–2015 and 2016–2017 were neither exceptionally energetic nor significantly different from other years. Anomalies of surface Eddy Kinetic Energy (EKE) calculated from the satellite altimetry show positive anomalies during 2014–2015 and negative anomalies during 2016–2017 (Supplementary Fig. S1). These anomalies are comparable to other years and therefore cannot explain the anomalous chlorophyll-*a* during 2014–2015 and 2016–2017 periods. The vertical displacement of thermocline induced by mesoscale eddies can modulate surface chlorophyll with the shallower thermocline associated with a cyclonic eddy leads to enhanced primary productivity by bringing high nutrient waters up into the euphotic zone^14^. The depth of 20 °C isotherm, a proxy for depth of thermocline, was slightly deeper (10–20 m) than the climatology during both periods and do not support a shallower nutricline, which co-occurs with thermocline, (Supplementary Fig. S1) and hence the observed year-to-year variations of the chlorophyll-*a*. This is consistent with the satellite altimetry derived sea level anomalies (Supplementary Fig. S1). Submesoscale eddies can generate areas of sharp horizontal density gradients which in turn lead to vertical restratification and promote patchy blooms (at spatial scales of 1–10 km and timescales of days). This mechanism has been proposed for phytoplankton blooms in the north Atlantic^15^ and in the north Atlantic subpolar gyre^16^ before the vernal restratification. Although such eddy-induced restratification is likely a contributor to the total productivity in the northern Arabian Sea, it alone cannot be accountable for significant chlorophyll-*a* differences between these periods. Furthermore, spatial scales of blooms presented here are much larger than the submesoscale scales of 1–10 km.

### Winter-mixing intermittency and ephemeral restratification in 2014–2015

**Figure 2:**
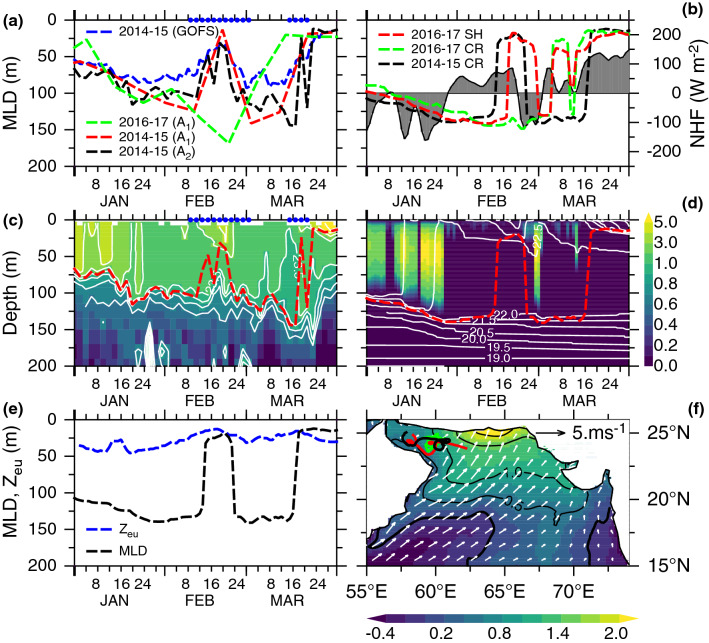
Winter-mixing intermittency and stratification during 2014–2015. (**a**) Mixed layer depths (MLD, m) from two along-track Argo observations (A_1_ and A_2_) in the northwestern Arabian Sea including the Gulf of Oman in winter (A_1_: red, A_2_: black) 2014–2015 (A_1_: green) 2016–2017, and (blue) box-averaged MLD from GOFS (58°–66° E, 18°–26° N) during 2014–2015. A_1_ (A_2_) had 10 (2)-day temporal resolution, Argo float tracks are shown in (**f**). (**b**) 1-D model MLD at 62° E, 25° N (closest to Argo float locations) from the control run (CR) during (black) 2014–2015 (green) 2016–2017, and (red) 2016–2017 specific humidity (SH) perturbation experiment. This experiment is designed to isolate the atmospheric forcing that caused 2014–2015 mixed-layer restratification by replacing 2016-2017 specific humidity with that during 2014-2015 while retaining all other forcing during 2016-2017 period. 1-D model net heat flux (NHF, W m^−2^) during 2014–2015 is shaded in grey. (**c**) Along-track Argo (A_2_) temperature profiles (°C, shaded with contours > 21 °C) in the upper 200 m, and MLD (red dashed line). (**d**) 1-D model temperature profiles (contours), MLD (red dashed line), and temperature diffusivity (shaded, 10^3^, cm^2^ s^−1^), proxy for turbulent mixing. (**e**) MODIS-Aqua derived daily euphotic depth centered around 1-D model location (61°–63° E, 24°–26° N) smoothed using 5-day boxcar filter (blue dashed line) compared to 1-D model MLD (black). (**f**) February 2015 monthly anomalies of vector wind (m s^−1^) and anomalies of specific humidity (g kg^−1^, shaded with contours) from the CFSR. Anomalies are relative to the 2011–2021 period. Blue circles in (**a,c**) mark the periods of mixed-layer restratification induced by atmospheric high-pressure systems. Note that Argo floats were confined to northwestern Arabian Sea including the Gulf of Oman while the largest specific humidity anomalies are located in the central northern Arabian Sea. Typical winter mixed layer evolution for 2016–2017 is included for comparisons.

In the northern Arabian Sea, in typical winter conditions the mixed layer starts to deepen in November, reaching a maximum depth during January–February (Fig. 2a) due to convective mixing driven by surface buoyancy loss^17,18^. In February 2015, along-track Argo float observations (A_1_ and A_2_) in the northwestern Arabian Sea (including the Gulf of Oman) revealed a period of anomalously shallow mixed layer depth (Fig. 2a). Between 11 and 20 February 2015, the mixed layer shoaled by 111 m, from 124 to 13 m. After deepening to 141 m on 2 March, it again attained a shallow depth of 19 m on 22 March (spring shoaling). In addition to the first shallow mixed layer event, a nearby Argo float (A_2_ with 2-day temporal resolution) indicated a second period of shallow mixed layer (24 m) during 17–19 March 2015, just before the spring shoaling. These winter-mixing intermittency events were not confined to the Argo float locations, but also broadly distributed over a wider area in the northern Arabian Sea as confirmed by the area averaged mixed layer depth from a global ocean data assimilative model (GOFS, Fig. 2a). The along-track Argo temperature profiles (A_2_) showed concurrent ephemeral restratification during these periods with temperature in the upper 50 m increased by 0.5 °C (24° to 24.5 °C; Fig. 2c). The erosion of ephemeral restratification due to winter cooling (between shallow mixed layer periods) caused the mixed layer to deepen until late March when the spring warming initiated the onset of vernal restratification.

A one-dimensional (1-D) ocean model simulation, which complements the observational evidence, successfully replicated the features of winter-mixing intermittency, including the timing of ephemeral and vernal restratification, surface warming (0.5 °C), and mixed layer shoaling during 2014–2015 winter (Fig. 2b,d). Between 14 and 21 February, the mixed layer shoaled abruptly from 119 to 19 m, followed by rapid deepening to 128 m on 25 February which persisted until 19 March when the mixed layer shoaled to 17 m. Because of this agreement, the dominant factors driving the mixed layer restratification can be elucidated from the surface heat flux, which is a balance between incoming short-wave radiation, long-wave radiation and sensible and latent heat fluxes. The region experienced a net heat gain by the ocean during 29 January–21 February (~ 50 W m^−2^, Fig. 2b). It was during this period the mixed layer stratification started to increase (Fig. 2d). Despite heat gain by the ocean the mixed layer remained deep (139–119 m) until 14 February. A closer examination revealed that the mixed-layer stratification occurred when net heat flux steadily increased from 23 to 86 W m^−2^ over the 10–20 February period with a time-lag of ~ 4 days. Subsequent heat loss (− 88 W m^−2^) during 22 February–2 March eroded the mixed layer stratification (time-lag of 4 days) resulting in deep mixed layer depth (140 m). Mixed layer remained deep until 18 March (despite heat gain by the ocean) when heat flux rapidly increased from 6 to 117 W m^−2^ (14–18 March). This triggered the onset of vernal restratification.

Mixed layer restratification events require sufficiently long calm periods with a relaxation of atmospheric forcing as the restratification mechanisms compete with vertical mixing induced by surface buoyancy loss^15,16,19–22^. In the northern Arabian Sea, model sensitivity experiments with prescribed high specific humidity have shown to suppress the winter heat loss and convective mixing^18^. We therefore postulate that a reduction in latent heat flux, aided by high specific humidity, can be ascribed to the surface heat gain during the mixed layer restratification periods. Anomalies of specific humidity in the northern Arabian Sea in February 2015 were 9.5% higher than the climatological mean, indicating anomalously high humid conditions (Fig. 2f). Anomalies of specific humidity progressively increased from 0.5 to 1.5 g kg^−1^ north of 20° N with the largest values occurring in the central northern Arabian Sea. Furthermore, anomalies of surface wind vectors exhibited predominantly southwesterly winds in February 2015 (Fig. 2f), supporting the idea that the abnormally high humidity air originated farther south or southwest. These anomalous conditions contrasted typical winter conditions when prevailing northeasterly winds (Supplementary Fig. S2) bring relatively cool, dry continental air (low humidity) from north^6,17^.

A 1-D model perturbation experiment confirms that the ephemeral restratification in February 2015 was triggered by buoyancy gain resulting from the specific humidity increase. By replacing specific humidity during 2016–2017 with 2014–2015 period (and all other 2016–2017 atmospheric forcing retained) in a 1-D model experiment, we replicated the ephemeral restratification and associated mixed layer shallowing event during February 2017, resembling that during February 2015 (Fig. 2b). Specifically, the duration of shallow mixed layer (10 days) exactly matches that during February 2015 despite a time lag of 4 days response. This stems from the fact that heat gain during early February 2015 played a key role during the preconditioning period of the mixed-layer restratification. Thus, both the anomalies of specific humidity and winds and the 1-D model experiment support the hypothesis that warm humid air, aided by anomalous southwesterly winds, exacerbated the heat gain by the ocean and drove the transient mixed layer restratification in February 2015.

### Ephemeral restratification induced by atmospheric high-pressure systems

**Figure 3:**
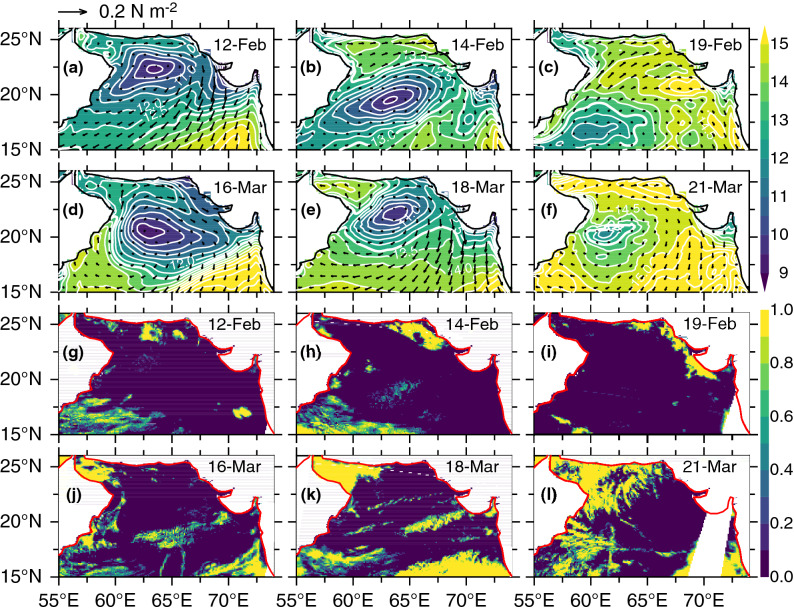
Winter-mixing intermittency triggered by atmospheric high-pressure systems. Mean daily CFSR specific humidity (g kg^−1^) and wind-stress (N m^−2^) for (**a**) 12 February, (**b**) 14 February, (**c**) 19 February, (**d**) 16 March, (**e**) 18 March, (**f**) 21 March, 2015 encompassing the periods of two mixed-layer restratification events. MODIS-Aqua daily cloud fraction during (**g**) 12 February, (**h**) 14 February, (**i**) 19 February, (**j**) 16 March, (**k**) 18 March, (**l**) 21 March, 2015. The scale 0 to 1 indicates clear sky to fully cloudy conditions with white areas indicating land or no data. The southwesterly winds to the west of high-pressure systems transport warm humid air to the northern Arabian Sea leading to humidity increase, while northeasterly winds to the east bring cold dry air from the north, although its impact is confined to a small region in the eastern Arabian Sea.

Analyses of specific humidity and wind-stress during February–March 2015 revealed that the observed restratification events were triggered by the atmospheric high-pressure systems. A high-pressure system, an anticyclonic atmospheric circulation (unlike a cyclonic atmospheric low), is characterized by clear skies, light winds and creates a stable, calm weather over the ocean it covers. During February–March 2015, we identified two high-pressure systems, associated with time scales of several days to weeks, that created conditions conducive to higher humidity in the northern Arabian Sea (Fig. 3). A slow-moving high-pressure system (12–23 February 2015), which stretched zonally across the entire northern Arabian Sea, appeared on 12, slightly intensified around 14, remained near-stationary during 19–23, and dissipated on 24 February. A similar, but near-stationary, atmospheric high occurred during 16–23 March 2015. Associated with these atmospheric high was the winds rotating clockwise with westerly to southwesterly to the north of 20° N. As these atmospheric high-pressure systems progressed south and southeast (or remained near-stationary), the southwesterly winds to the west of high-pressure system transported warm and humid air from the south to the northern Arabian Sea. As a result, the specific humidity in the northern Arabian Sea increased in excess of ~ 13 g kg^−1^ (Fig. 3). Specifically, these high-pressure systems during these events accounted for 35% and 52% specific humidity increase north of 20° N respectively. The high humidity led to reduction in latent heat flux (due to smaller air-sea humidity difference) resulting in heat gain by the ocean, which is consistent with the 1-D model results (Fig. 2b,d). The increased heat gain caused surface warming and generated a stably stratified upper ocean (Fig. 2c,d). Thus, the mixed layer intermittency observed during both periods, 15–24 February and 18–23 March 2015 (Fig. 2c), were driven directly by these high-pressure systems, although 1-D model failed to distinguish the latter from the spring warming.

An atmospheric high is often accompanied by clear skies as the divergence of descending cool air disperse clouds. Satellite derived daily cloud cover during both mixed layer restratification periods revealed clear sky conditions in the northern Arabian Sea representing below 10% fractional clouds. During February 2015 mixed layer restratification, the northern Arabian Sea including the Gulf of Oman experienced clear sky conditions except a few patchy areas near the coast (Fig. 3g-i). Daily cloud cover during the vernal restratification period (March 2015) exhibited similar conditions except in the northwestern Arabian Sea including the Gulf of Oman where fully cloud conditions persisted (Fig. 3j–l). The clear sky conditions reinforced the critical role of solar heating at the ocean surface which not only inhibited winter cooling and convective mixing but also favored phytoplankton growth aided by sunlight.

The high-pressure systems characterized by clear sky conditions are expected to bring more short-wave radiation. We performed additional 1-D model perturbation experiments to identify and isolate the role of each component of the atmospheric forcing including the short-wave heat flux on the mixed layer restratification. The experiment with perturbed short-wave flux failed to reproduce mixed layer shoaling. A lack of mixed layer response in the 1-D model experiment likely indicate bias in the CFSR reanalysis short-wave flux, which is sensitive to how cloud cover is estimated in the global weather and climate prediction models. The area averaged (58°–66° E, 18°–26° N) daily mean CFSR short-wave heat flux during January–March 2015 confirms the lack of short-wave flux increase during the passage of an atmospheric pressure system in February 2015 (Supplementary Fig. S3). The short-wave flux progressively increased from 220 to 250 W m^−2^ during February 2015, which is negligibly small to have an appreciable impact on mixed layer restratification.

### Response of chlorophyll-*a* to ephemeral restratification events

**Figure 4:**
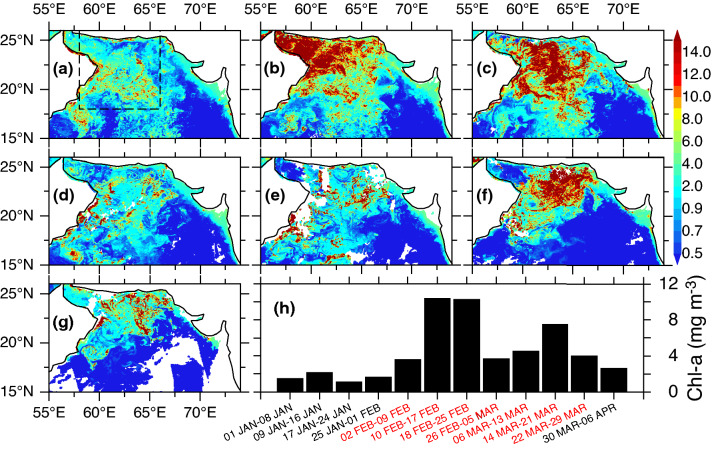
Anomalous chlorophyll-*a* blooms triggered by atmospheric high-pressure systems. MODIS-Aqua 8-day chlorophyll-*a* (mg m^−3^) during (**a**) 2–9 February, (**b**) 10–17 February, (**c**) 18–25 February, (**d**) 26 February–5 March, (**e**) 6–13 March, (**f**) 14–21 March, (**g**) 22–29 March, 2015 and (**h**) Area-averaged chlorophyll-*a* histograms (58°–66° E, 18°–26° N, Fig. 4a, x-axis denotes 8-day composite time window; red axis-labels correspond to spatial maps in (**a–g**)) during January–March 2015.

Satellite-based 8-day chlorophyll-*a* concentration peaked twice during the boreal winter of 2014–2015 coinciding almost exactly with the timing of the ephemeral and vernal restratification events triggered by atmospheric high-pressure systems (Fig. 4). Large areas of high chlorophyll-*a* concentration in excess of 10 mg m^−3^ first appeared in the northwestern Arabian Sea including the Gulf of Oman during 10–17 February (Fig. 4b). This period coincided with the high specific humidity > 13 g kg^−1^ and clear-sky conditions (Fig. 3) both exacerbated mixed layer restratification by promoting ocean heat gain. The spatial distribution of chlorophyll-*a* shifted farther to the east in the following week (18–25 February) with a marked reduction in the Gulf of Oman owing to stripped of nutrients by previous phytoplankton growth or losses due to consumption by zooplankton, respiration and sinking. Responding to ephemeral stratification, the chlorophyll-*a* between 2–9 and 10–17 February increased rapidly by 189% (3.6 to 10.4 mg m^−3^) and persisted through 18–25 February. After dropping to 2–9 February level, chlorophyll-*a* reached a secondary peak (7.5 mg m^−3^) on 14–21 March, a 50% increase from the week before (6–13 March), marking the onset of vernal stratification (Fig. 4h). The spatial distribution of chlorophyll-*a* during 14–21 March 2015 (Fig. 4f) was aligned with the atmospheric high-pressure system and cloud cover. In particular, relatively low chlorophyll-*a* concentration in the northwestern Arabian Sea including the Gulf of Oman can be attributed to the increased cloud cover (Fig. 3) despite high specific humidity. Relatively low chlorophyll-*a* concentrations between 26 February and 13 March (Fig. 4d,e) is consistent with the period of deep mixed layer (Fig. 2a,b). The subsequent phytoplankton bloom (14–21 March) reflects an increase in nutrient loads during the period when convective mixing was active. Thus, the chlorophyll-*a* during the ephemeral restratification event, which lasted twice as long, was ~ 39% higher than that during the vernal stratification, contributed to the unprecedented chlorophyll-*a* during February 2015 (Fig. 1a). A slight increase in chlorophyll-*a* during 2–9 February coincided with the positive heat flux preceding the ephemeral restratification. This is consistent with the modeling results in the northeast Atlantic^19–21^.

Phytoplankton blooms are created by an array of factors including sunlight, turbulent mixing, depth of euphotic zone and availability of nutrients, that can combine to cause a bloom in the northern Arabian Sea. Although availability of sunlight is not a limiting factor for phytoplankton growth in the tropics (especially during vernal restratification), the clear skies that accompanied the atmospheric high (Fig. 3) favored to trigger a bloom. Furthermore, light winds associated with high-pressure system led to a reduction in turbulent mixing thereby promoting phytoplankton growth by maintaining phytoplankton cells in the euphotic zone, in accord with the critical turbulence hypothesis^20,21,23^. The average wind speed during these events was 3.5 and 4.1 m s^−1^ respectively, which is too weak to induce any appreciable turbulent mixing. The temperature diffusivity, a proxy for vertical mixing intensity, was negligibly small during the period of high-pressure systems (Fig. 2d) further supporting the claim that turbulent mixing was significantly suppressed. Although typical winter mixed layer depth in the northern Arabian Sea (e.g. 50–150 m in 2017) is significantly deeper than the euphotic depth, the mixed layer during the restratification periods attained shallower than the euphotic depth, a condition that is necessary to initiate phytoplankton blooms according to the critical depth paradigm^24^. The satellite derived euphotic depth, which ranged between 44 and 13 m, gradually shoaled from 39 to 21 m during January–February followed by a slight deepening to 24 m in March 2015 (Fig. 2e). For both periods, the 1-D model mixed layer depth was shallower than or almost equal to the euphotic depth with Argo float indicating a shallowest mixed layer depth of 13 m during the first restratification event. Conversely, the primary productivity was suppressed when mixed layer was deeper than the euphotic depth, consistent with recent findings in the northeastern Arabian Sea^25^. The fact that these independent observations and model results fit into a consistent scenario provided confidence in our results.

### Calm weather as a precursor to ocean productivity

**Figure 5:**
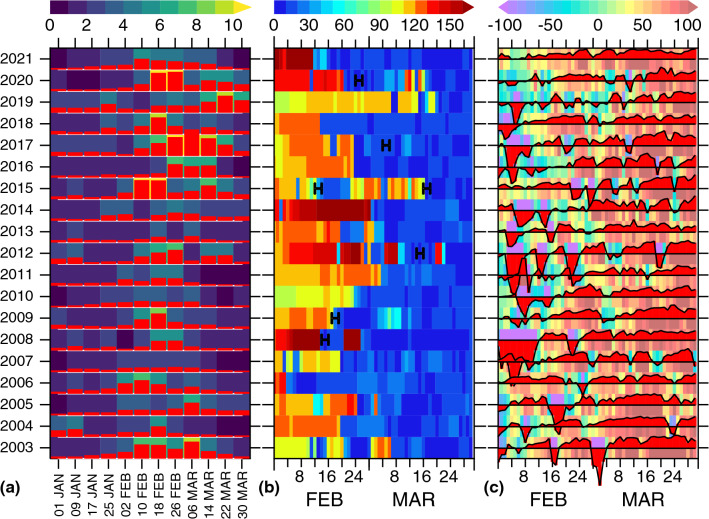
Linking year-to-year chlorophyll variations to ephemeral restratification. (**a**) MODIS-Aqua 8-day chlorophyll-*a* concentration (mg m^−3^, 58°–66° E, 18°–26° N) during January–March (x-axis denotes 8-day time range with 1-Jan indicating 1–8 January). 1-D model daily mean (**b**) mixed layer depth (m, based on the criteria density increase from the surface by 0.125 kg m^−3^) and (**c**) net-heat flux (W m^−2^) at 62° E, 24° N for the period 2003–2021 using interannual CFSR forcing. For clarity, chlorophyll and net-heat fluxes are also depicted as histograms (red). Positive (negative) heat flux indicates heat gain (loss) by the ocean. Periods of selected winter-mixing intermittency triggered by high-pressure systems (confirmed by CFSR specific humidity, Supplementary Fig. S4) are denoted with a letter ‘H’. 1-D model simulations at 62° E, 24° N broadly captures the mixed layer response to atmospheric high-pressure systems that traverse the northern Arabian Sea, so qualitative comparison between mixed layer depth, net-heat flux and chlorophyll-*a* is possible.

**Figure 6:**
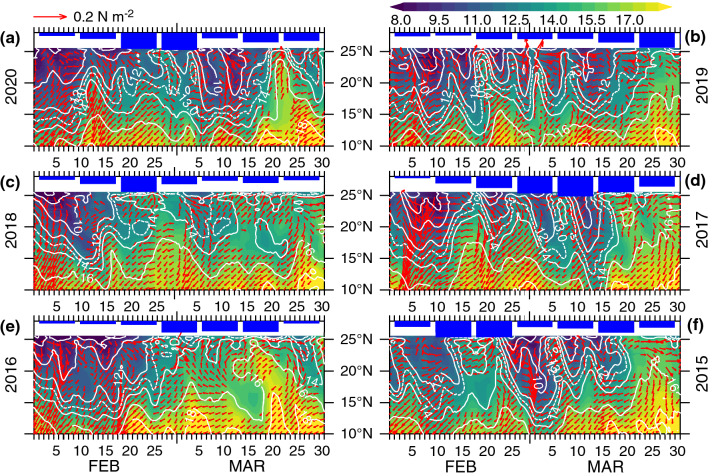
Representative atmospheric high-pressure systems. Time-latitude plot of CFSR specific humidity (58°–70° E) and wind-stress (62° E, wind-stress > 0.025 N m^−2^ are plotted) during February–March, (a–f) 2020–2015. Hourly fields are smoothed using a 48-h Boxcar filter. MODIS-Aqua 8-day chlorophyll-*a* observations are plotted as histograms on the top. Northward penetration of 13 g kg^−1^ specific humidity (dashed contour) in phase with the southwesterly winds north of 20°N (characterized the high-pressure systems) correlated well with the weekly chlorophyll fluctuations and peaks.

The ephemeral restratification observed during February–March 2015 was not an isolated event, but appeared to be a recurrent feature every year with varying frequency and duration mediated by atmospheric high-pressure systems. We identified a number of pronounced restratification events (expressed as those periods in which the mixed layer shallower than 40 m; typical euphotic depth, lasting longer than 5 days during February–March) from 1-D model simulations during 2003–2021 period (Fig. 5b). Notable periods of restratification include 2003 (8 days), 2008 (7 days), 2009 (16 days), 2012 (6 days), 2015 (10 days), 2017 (5 days) and 2020 (18 days). Furthermore, the specific humidity and winds pattern can be linked these events to the atmospheric high-pressure systems (Supplementary Fig. S4). These events are characterized by exacerbated heat gain (Fig. 5c) aided by increased specific humidity (Fig. 6) and concurrent elevated chlorophyll-*a* concentrations (Fig. 5a). Responding to mixed layer restratification in 2020 for example, the chlorophyll-*a* increased abruptly by 160% (3.86 to 10.04 mg m^−3^) between 10–17 and 18–25 February, and persisted through 26 February–5 March. A secondary chlorophyll-*a* peak, albeit 44% weaker than the first event, occurred during 14–21 March coinciding with the vernal stratification.

A further analysis of specific humidity, however, revealed that the high-pressure systems exerted a major control not only on the ephemeral restratification but also on the initiation and maintenance of vernal restratification and the ensuing productivity (Fig. 6). The onset of vernal restratification, which is primarily driven by increased solar radiation, coexisting with atmospheric high-pressure systems (as is the case during March 2015) appeared to accelerate the restratification processes. The onset of vernal stratification and concurrent chlorophyll-*a* peak during 18–25 February 2018 (9.3 mg m^−3^, a 103% increase from a week before) among other periods, and a secondary chlorophyll-*a* peak during 14–21 March 2020, coincided with the periods of high humidity (> 13 g kg^−1^) and southwesterly winds north of 20° N (Fig. 6), both are characteristics of an atmospheric high. The responses of chlorophyll-*a* to mixed layer stratification events were similar during other periods except differences in intensity and timing of the blooms.

Restratification process typically involves sufficiently long periods of heat gain under light wind conditions. Therefore, not all high-pressure systems necessarily lead to prolonged mixed layer restratification as some systems progress (or dissipate) rather quickly causing rapid fluctuations in surface heat flux that are too fast for mixed layer to respond. The surface heat flux in 2019, for example, fluctuated rapidly between heat gain and loss while maintaining a mixed layer depth of ~ 100 m until prolonged ocean heat gain occurred in late March 2019 (Fig. 5). These fluctuations occurred concurrently with the variations in humidity on the same time scale, as indicated by the northward intrusion of a 13 g kg^−1^ contour and the southwesterly winds north of 20° N (Fig. 6). These episodic warming resulted a weakly stratified water column while subsequent cooling de-stratified and deepened the mixed layer. Consequently, the phytoplankton blooms maintained over a longer period of time aided by resupply of nutrients up into the euphotic zone by convective mixing between the calm periods. The episodic restratification events between 18 February and 13 March 2019 contributed to the elevated levels of chlorophyll-*a* (Fig. 6). Similarly, periodically interrupted convective mixing triggered by calm periods during late February–March 2017 caused anomalously high chlorophyll-*a* during March 2017 (Figs. 1, 6). In summary, the timing, frequency and duration of the atmospheric highs exert a large control on primary productivity in the northern Arabian Sea.

## Discussion

During winter, the dry, cool air brought by prevailing northeasterly trade winds leads to surface ocean heat loss and convective mixing in the northern Arabian Sea^17,18^. The current paradigm is that the convective mixing process leads to the injection of nutrients up into the surface waters and exert a dominant control on winter productivity^6^. However, this mechanism cannot entirely explain the large year-to-year variations of the winter productivity (Fig. 1a). Here we show observational evidence for widespread winter productivity triggered by intermittent mixed layer restratification mediated by atmospheric high-pressure systems. As these weather systems with clockwise circulation move south, the southwesterly winds to the west of high-pressure system transport warm, humid air from south to the northern Arabian Sea resulting in an increase in specific humidity. This reduces the latent heat flux thereby increasing the surface heat gain by the ocean. This combined with abundant sunlight aided by clear skies, reduced turbulent mixing due to weak winds inhibits vertical mixing thereby increasing the residence time of phytoplankton cells in the euphotic layer, allowing a bloom to develop. In particular, the anomalously high phytoplankton blooms (twofold increase) in recent winters of 2014–2015 and 2016–2017 are linked to atmospheric high-pressure systems.

Although we demonstrated primarily the physical control of the primary productivity in this region, both biological and chemical factors play important roles and should not be overlooked^25,26^. We further note that primary productivity as identified here is as seen from the satellite and it is important to discern that from the high levels of subsurface chlorophyll-*a* maximum that has been observed in the Arabian Sea^6,27,28^. During April–May, a subsurface chlorophyll maximum was observed in the northern Arabian Sea associated with the nutricline^6^, and in the northeastern Arabian Sea during March^28^ using Bio-Argo floats while a persistent maximum was reported between March 2010 and 2011 in the southeastern Arabian Sea^27^. In the southeastern Arabian Sea, westward propagating Rossby waves lead to vertical fluxes of chlorophyll from the subsurface chlorophyll maximum and contributed significantly to mixed layer blooms^27^. However, such a subsurface chlorophyll maximum was less pronounced particularly in the north in winter as higher values from the surface progressively declined with depth^6,25^. Therefore, it is highly unlikely that the high surface chlorophyll observed during the mixed layer restratification periods (Fig. 4) resulted from the vertical displacement of subsurface chlorophyll maximum. Nevertheless, the chlorophyll-*a* values in excess of 10 mg m^−3^ reported here (Fig. 4) are much larger than that observed within the subsurface chlorophyll maximum reported in this region (1–2 mg m^−3^)^27,28^.

We ascribed the weekly chlorophyll-*a* fluctuations with multiple peaks to the ephemeral restratification events, whose onset and maintenance is closely linked to the timing, frequency, duration and intensity of atmospheric high-pressure systems that traverse the northern Arabian Sea every winter and spring. Similarly, primary productivity is also influenced by an array of factors including the availability of nutrients, sunlight and turbulence mixing. Although we related periods of high levels of chlorophyll-*a* to concurrent high-pressure systems during particular years, not all high-pressure systems likely lead to anomalous blooms, especially those fast-moving systems. However, the analyses of specific humidity indicated multiple high-pressure systems traversing northern Arabian Sea almost every year (Fig. 6) that can extend the phytoplankton growth. Our findings suggest winter mixing intermittency triggered by high-pressure systems contribute to primary productivity, albeit to a lesser extent in some years than other, and should be treated as a significant source for year-to-year chlorophyll-*a* variability in the northern Arabian Sea. In particular, the onset of spring bloom (peak chlorophyll-a concentration), which typically occurred in February of each year until 2012, shifted from February to March in the last decade (Fig. 1a). The frequency of spring bloom occurrence in March has increased since 2012 with chlorophyll-a during March exhibiting higher or closer to that in February with the exception of 2015. Consequently, the chlorophyll-*a* during March increased by 72% in the last decade (2012–2021) compared to the decade before (2003–2011) while that during January–February remained nearly flat (Fig. 1a). We argue that the anomalous persistent atmospheric highs, which are becoming more frequent during the last decade, can be attributed to the recent trends of extended phytoplankton growth into March. The characteristics of these systems can be influenced by the interhemispheric pressure gradients that govern the winter monsoon with recent reports suggesting declining pressure gradient under a warming climate^29^ or loss of snow cover over the Himalayan–Tibetan plateau region^30^. Since climate change is expected to increase the frequency and intensity of extreme weather events including marine heat waves^31^, it is possible that ephemeral stratification events during winter and early spring could become more frequent, expanding their role in global photosynthetic fixation and export of carbon from the surface ocean.

## Data and methods

### Data

We used version 4 of the Met Office Hadley Centre series of data sets (EN4.2)^32^ of global quality-controlled ocean temperature and salinity profiles and monthly objective analyses to provide baseline of mixed layer depth and 20 °C isotherm depth (D_20_). Sea level anomalies (SLA) and surface Eddy Kinetic Energy (EKE) products are derived from satellite altimetry and processed by SSALTO/DUCAS and distributed by AVISO+ (https://www.aviso.altimetry.fr) with support from CNES. To delineate the atmospheric forcing that lead to the winter mixing intermittency, we employed National Centers for Environmental Prediction (NCEP) Climate Forecast System Reanalysis (CFSR) data^33^ during the period 1998–2021. In particular, we used specific humidity and winds as proxies to identify the atmospheric high-pressure systems. Net heat flux is computed from solar and long-wave radiation, latent and sensible heat fluxes using CFSR meteorological fields. Anomalies are calculated from the 1998–2021 mean. It should be noted that same dataset is being used to force the 1-D model simulations.

We examined individual Argo float observations to identify transient restratification events in the northern Arabian Sea. Two Argo float observations (ID 2901860 and 2901476) during 2014–2015 and 2016–2017 were used; former Argo sampled every 10 days and latter every 2 days. The 2-day temporal resolution enabled us to identify two mixed-layer stratification events in 2015.

This study also utilized satellite derived observations of chlorophyll-*a* concentration, fractional cloud cover and euphotic depth. Monthly mean and 8 days composites of chlorophyll-*a* concentration (mg m^−3^) obtained from the MODIS-Aqua sensor (Moderate Resolution Imaging Spectroradiometer) with spatial resolution 4 km were downloaded from the NASA web server (https://oceandata.sci.gsfc.nasa.gov/MODIS-Aqua) covering the period January 2003–December 2021. The daily composites of fractional cloud derived from MODIS-Aqua during February–March 2015 is downloaded from https://neo.gsfc.nasa.gov/ provided by the MODIS Atmosphere Science Team, NASA Goddard Space Flight Center. The euphotic depth (Z_eu_)^25^ using the diffuse attenuation coefficient of downward irradiance at 490 nm with a spatial resolution of 4 km obtained from MODIS-Aqua (https://hermes.acri.fr).

### Models

The numerical models, initial and boundary conditions used in this study are same as in Thoppil et al. ^12^. Briefly, in the absence of eddy induced re-stratification by advection, convective mixing can be approximated as a one-dimensional (1-D) process, where surface buoyancy loss triggers convection with the buoyancy loss being partly compensated by fluxes at the mixed layer base^18^. In the northern Arabian Sea, this assumption is not untenable to the process under study because winter convection and formation of Arabian Sea High Salinity Water mass (ASHSW) have been reproduced using a 1-D model^18^. Here we use Hybrid Coordinate Ocean Model (HYCOM)^34^ configured as a 1-D model to simulate the upper ocean response to atmospheric forcing. The K-Profile Parameterization (KPP)^35^ vertical mixing scheme is used.

The 1-D model is forced with NCEP-CFSR hourly fields of wind-stress, solar radiation, specific humidity, air-temperature and precipitation. Model SST is used to calculate the surface heat flux. All 1-D model simulations are re-initialized in May from the Generalized Digital Environmental Model climatology (GDEM4)^36^ temperature and salinity climatology, and integrated for a year (May–April) using realistic atmospheric forcing. Thus, the model fields evolve entirely due to year-to-year variations in the atmospheric forcing. There are 241 vertical layers with 1 m grid spacing in the upper 200 m, and the remaining 40 layers are distributed between 20 and 1000 m with variable grid spacing. As the deepest mixed layer during the period is shallower than 150 m, the coarse resolution below 200 m has negligible impact on the evolution of thermohaline properties. 1-D model simulations are performed at two locations (62° E, 25° N and 62° E, 24° N) in the northern Arabian Sea using the atmospheric forcing spanning from 2003 to 2021, with a special focus on 2014–2015 winter.

The 1-D model is complimented by results from a 1/12.5° Global Ocean Forecast System (GOFS), which is a global, three-dimensional (3-D) HYCOM data assimilative system^37^. The GOFS consists of HYCOM for the ocean and the Navy Coupled Ocean Data Assimilation (NCODA), which uses a 3-D Variational Data Assimilation (3-DVar) method for assimilation of ocean observations. The operational GOFS uses atmospheric forcing from the operational NAVy Global Environmental Model (NAVGEM).

## Supplementary Information

Supplementary Figures.

## Data Availability

The Met Office Hadley Centre series of datasets (EN4.2) of global quality-controlled ocean temperature and salinity profiles used in this study are openly available^32^. Argo float observations are publicly available at argo.ucsd.edu. SLA and EKE product are available at www.aviso.altimetry.fr. The NCEP CFSR data are openly available and can be obtained from rda.ucar.edu. HYCOM reanalysis product used in this study can be accessed from www.hycom.org. The 1-D model output is available upon reasonable request to corresponding author.
