# Supplementary material for: Enhanced phytoplankton bloom triggered by atmospheric high-pressure systems over the Northern Arabian Sea
Source: Sci Rep. 2023 Jan 14;13:769. doi: 10.1038/s41598-023-27785-z (PMC9840610; doi:10.1038/s41598-023-27785-z)
Supplement: Supplementary file 1 — Supplementary Figures. [file 41598_2023_27785_MOESM1_ESM.pdf]

Supplementary Figures.

Enhanced Phytoplankton Bloom Triggered by Atmospheric high-pressure systems Over the Northern Arabian Sea

Prasad G. Thoppil*
Ocean Sciences Division
Naval Research Laboratory
Stennis Space Center, MS 39529, USA.

Revised and Submitted to the Scientific Report

*Corresponding author: Prasad Thoppil, Ocean Sciences Division, Naval Research Laboratory, Stennis Space Center, MS 39529, USA, Email: prasad.thoppil@nrlssc.navy.mil; Phone: 228-688-5500.

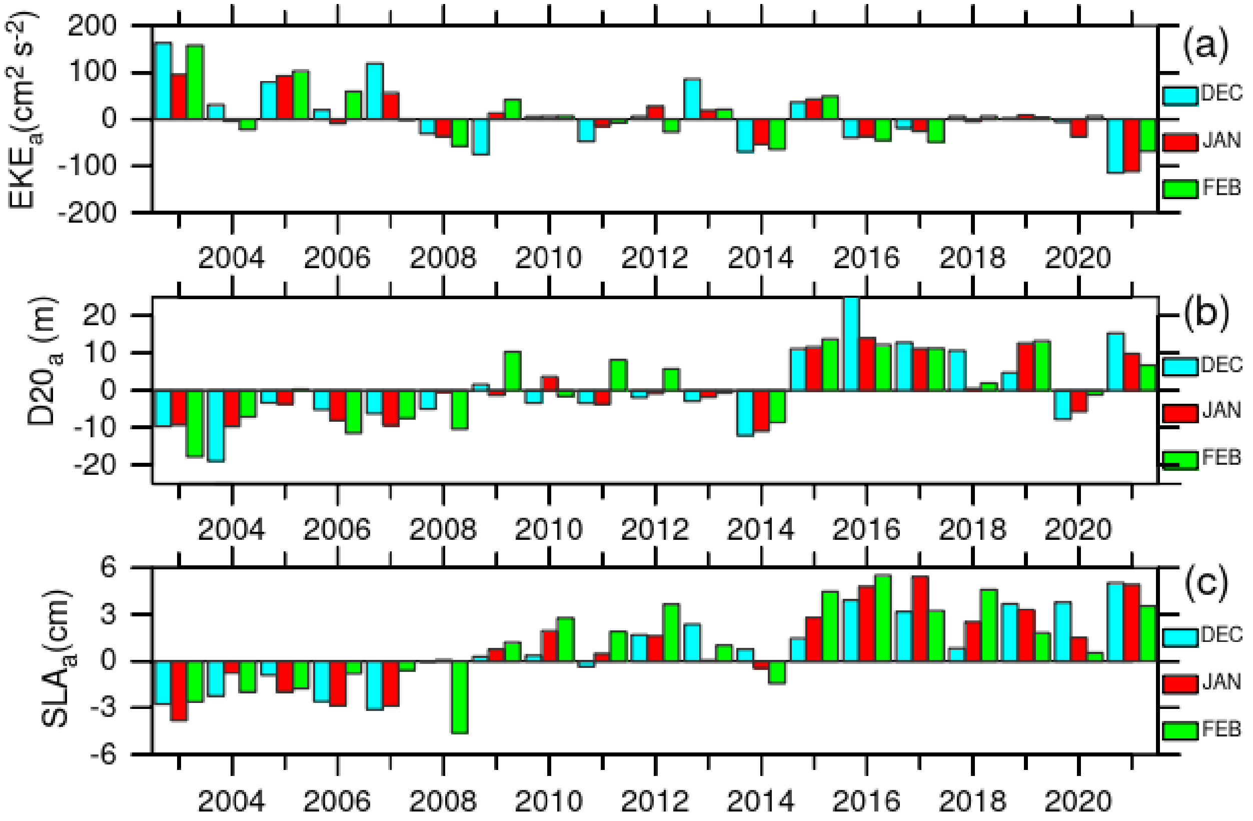

Fig. S1. (a) Monthly mean anomalies of surface Eddy Kinetic Energy (EKE, cm^2 s^{-2}) derived from the satellite altimetry suggest positive EKE anomalies during 2014-15 and negative anomalies during 2016-17. The fact that the EKE anomalies during the anomalous chlorophyll-*a* concentration are not exceptionally high and comparable to other periods preclude the possibility that anomalous chlorophyll-*a* are generated through eddy-induced processes. (b) Monthly mean anomalies of depth of 20°C isotherm (D20, m) calculated from the EN4.2 temperature profiles. The depth of thermocline, proxy for location of nutricline, an ocean layer with high variation of nutrient content which co-occurs with thermocline, do not support high chlorophyll-*a* concentration during winters of 2014 – 15 and 2016 – 17. (c) Sea level anomalies (SLA, cm) derived from satellite altimetry are consistent with D20 anomalies. Anomalies are computed from the 1998 – 2021 mean and averaged over 58° - 66°E, 18° - 26°N.

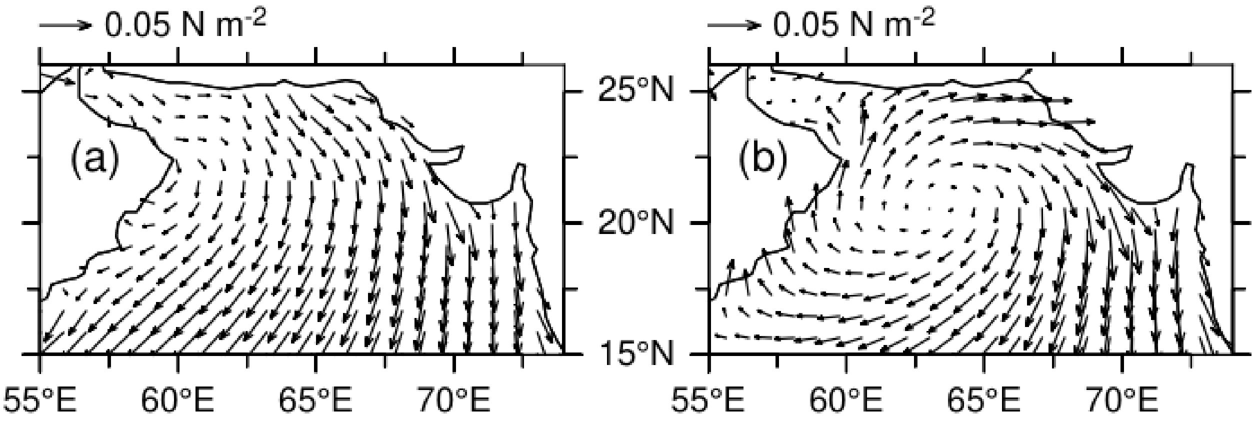

Fig. S2. (a) Climatological wind-stress (N m^{-2}) for February and (b) composite wind-stress associated with the atmospheric high-pressure events during 2015 – 2020. Climatology is relative to 2011 – 2021 period. During winter, winds are predominantly northeasterly which carry cold and dry continental air to the northern Arabian Sea triggering winter cooling and convective mixing. During the passage of atmospheric high-pressure systems, the winds north of ~20°N become southwesterly which bring warm and humid from the south to the northern Arabian Sea, leading to buoyancy gain and suppression of convective mixing.

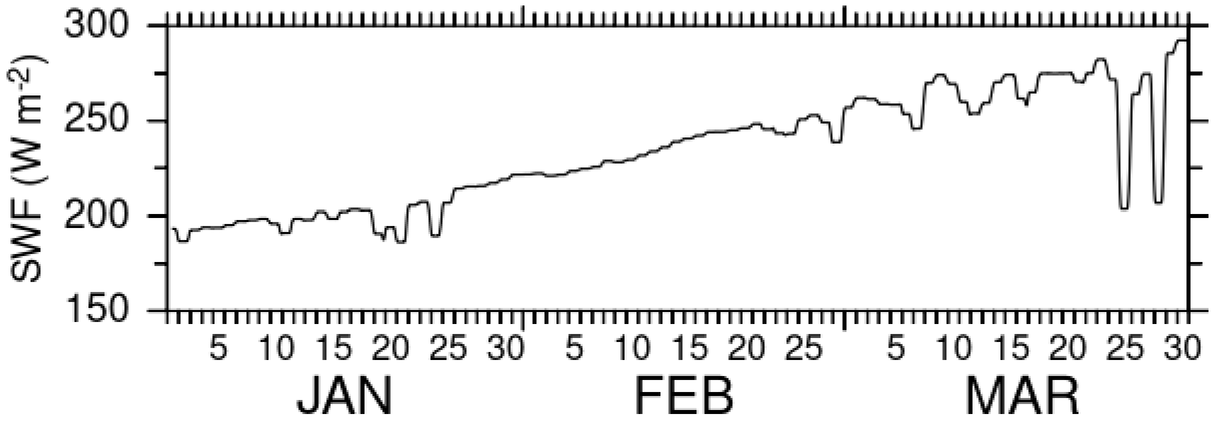

Fig. S3. Time-series of box averaged (58° - 66°E, 18° - 26°N) daily mean CFSR short-wave heat flux (W m^{-2}) during January – March 2015. The atmospheric high-pressure systems characterized by clear sky conditions could lead to increased incoming short-wave heat flux and thereby contributing to mixed layer restratification in addition to specific humidity driven latent heat flux. A 1-D model perturbation experiment with 2014 – 15 short-wave heat flux, while retaining all other forcing from 2016 – 17 period, failed to reproduce the mixed layer shoaling event. We attribute this to the lack of solar radiation peak associated with the high-pressure systems during February 2015 in the CFSR reanalysis product. The short-wave heat flux during February 2015 progressively increased from 220 to 250 W m^{-2}, which is negligibly small to have an appreciable impact on mixed layer restratification.

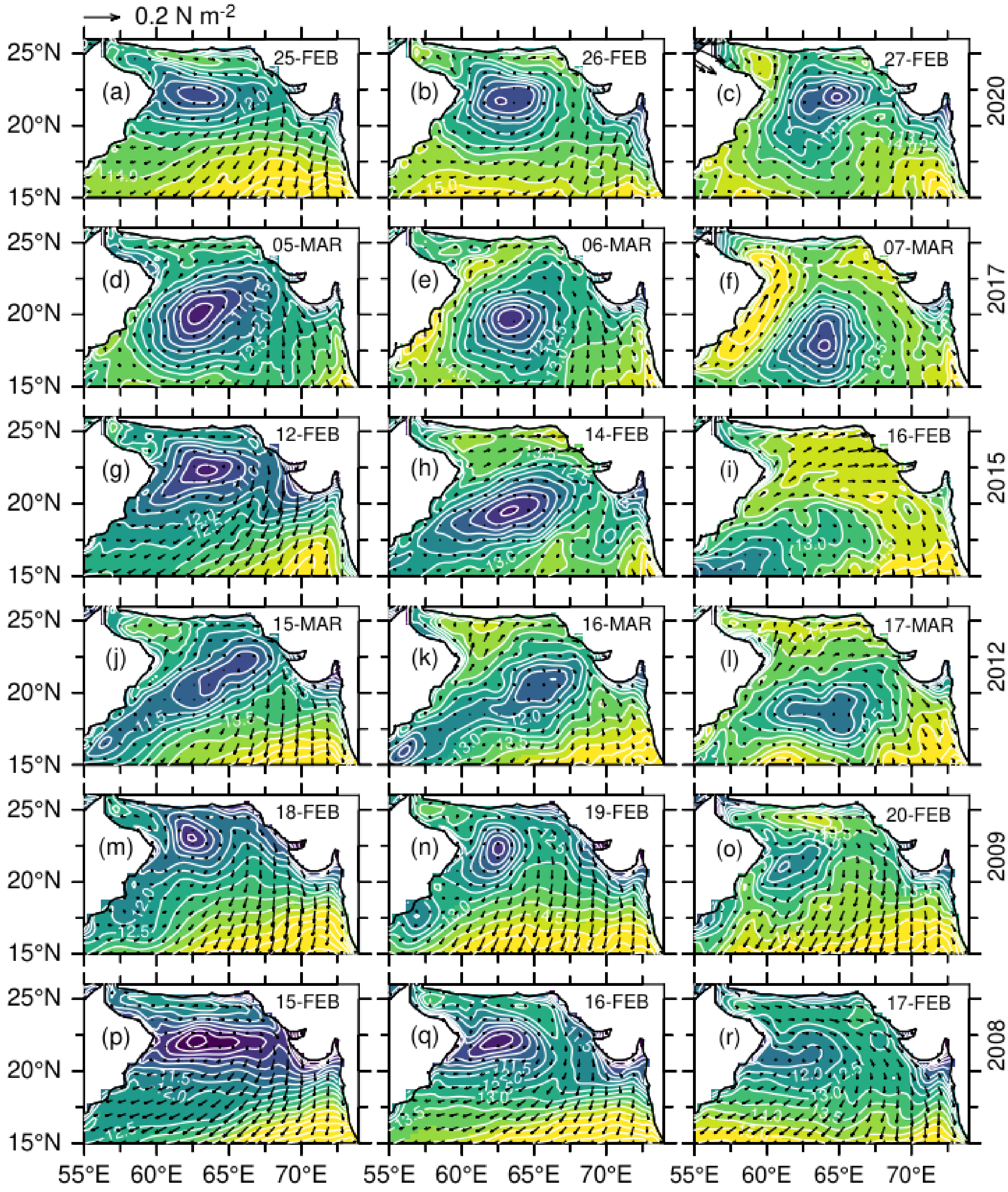

Fig. S4. Representative periods of atmospheric high-pressure systems. Mean daily CFSR specific humidity (g kg^{-1}) and wind-stress (vectors, N m^{-2}) for selected winter-mixing intermittency periods induced by atmospheric high-pressure systems. As the high-pressure systems progress southward into northern Arabian Sea, the winds north of ~20°N change from northeast to southwest. The southwesterly winds transport relatively warm and humid air to the north resulting in an increased

specific humidity. The increased humidity in turns decreases the latent heat flux thereby exacerbate the heat gain by the ocean. As a result, mixed layer stratifies and thereby promotes phytoplankton growth, supplemented with clear skies and reduced turbulent mixing aided by the high-pressure systems.
